# Efficacy of Pgp3 vaccination for *Chlamydia* urogenital tract infection depends on its native conformation

**DOI:** 10.3389/fimmu.2022.1018774

**Published:** 2022-11-16

**Authors:** Bo Peng, Shufang Zhong, Yaoqin Hua, Qizheng Luo, Weilei Dong, Chuan Wang, Zhongyu Li, Chunfen Yang, Aihua Lei, Chunxue Lu

**Affiliations:** ^1^ Institute of Pathogenic Biology, Hengyang Medical College, University of South China, Hunan Provincial Key Laboratory for Special Pathogens Prevention and Control, Hengyang, China; ^2^ Department of Pathology, Hengyang Medical College, University of South China, Hengyang, China; ^3^ Department of Obstetrics and Gynecology, The First Affiliated Hospital of University of South China, Hengyang, China

**Keywords:** *Chlamydia trachomatis*, Pgp3, vaccine, conformation structure, histopathology

## Abstract

Urogenital tract infections with *Chlamydia trachomatis* have frequently been detected among patients diagnosed with sexually transmitted infections, and such infections lead to inflammatory complications. Currently, no licensed chlamydial vaccine is available in clinical practice. We previously reported that immunization with recombinant *C. trachomatis* plasmid-encoded virulence factor Pgp3 provided cross-serovar protection against *C. muridarum* genital tract infection. Because Pgp3 is a homotrimer and human antisera only recognize the trimeric form of Pgp3, we compared the effects of the native conformation of Pgp3 (trimer) and heat-denatured Pgp3 (monomer) to determine whether the native conformation is dispensable for the induction of protective immunity against chlamydial vaginal challenge. Both Pgp3 trimer and monomer immunization induced corresponding specific antibody production, but only trimer-induced antibody recognized endogenous Pgp3, and trimer-immunized mouse splenocytes showed the highest IFN-γ production upon restimulation with the chlamydial elementary body or native Pgp3 *in vitro*. Importantly, only Pgp3 trimer-immunized mice showed shortened lower genital tract chlamydial shedding and decreased upper genital tract pathology. Thus, Pgp3-induced protective immunity against *Chlamydia* urogenital tract infection is highly dependent on the native conformation, which will guide the design of Pgp3-based polypeptides and multi-subunit chlamydial vaccines.

## Introduction

*Chlamydia trachomatis*, a gram-negative intracellular bacterium, comprises at least 19 serotypes. Serovars A to C are associated with ocular epithelial cell infection, serovars D to K primarily infect urogenital epithelial cells, and serovars L1 to L3 mainly target the lymphatic tissue (1). All *C. trachomatis* organisms share a conserved biphasic life cycle, switching between an infectious elementary body (EB) and a noninfectious reticulate body (RB). Chlamydial infection is initiated when the EB invades the host epithelial cells. The internalized EB rapidly differentiates into metabolically active RB. It replicates and differentiates back into EBs, which are released at a late stage of the life cycle and infect adjacent cells (2). Urogenital tract infections with *C. trachomatis* have frequently been detected among patients diagnosed with sexually transmitted infections, with an estimated annual incidence of over 131 million cases worldwide in 2018 (3). Although *Chlamydia* is sensitive to antibiotic treatment, most infected women are asymptomatic and easily overlooked, resulting in the spread of the infection and the occurrence of complications. Of note, 15%–40% of women with vaginal chlamydial infection develop inflammatory complications, including fallopian tube fibrosis and blockage, adhesion, ectopic pregnancy, and infertility (4, 5). The heavy social burden of chlamydial genital tract infection underscores the urgent need to develop an effective vaccine (6).

*C. muridarum* is a murine variant of *Chlamydia* and a model pathogen for studying the pathogenesis of *C. trachomatis*. *C. muridarum* vaginal infection induces hydrosalpinx in mice, similar to female fallopian tube infertility (7). Pgp3, a cryptic plasmid-encoded protein, has been implicated in chlamydial pathogenesis since Pgp3-deficient *C. muridarum* failed to induce hydrosalpinx after vaginal infection and could not colonize the gastrointestinal tract following oral inoculation (8, 9). Pgp3 is an ~84 kDa homotrimer consisting of a globular N-, tumor necrosis factor-resembling C-, and triple-helical coiled-coil M-domain (10). *C. muridarum* mutants deficient in any domain of Pgp3 attenuated the induction of hydrosalpinx, indicating that structural integrity is essential for Pgp3 function (11). Furthermore, the Pgp3 trimer is distributed in both the chlamydial EB outer membrane and infected cell cytosol. Antisera collected from chlamydial genital-infected women predominantly recognized Pgp3 with a higher frequency than any other chlamydial immunodominant antigen, including chlamydial protease-like activity factor (CPAF), major outer membrane protein (MOMP), and polymorphic outer membrane protein, and human antisera only recognize the trimeric form of Pgp3 (12–14).

Despite extensive efforts, there is still no chlamydial vaccine available for humans. Pgp3 serves as a chlamydial virulence factor and immunodominant antigen and has been frequently reported in previous vaccine studies. Immunization of C3H/HeN mice with pgp3 DNA protected against the spread of *C. trachomatis* from the vagina to the upper genital tract (15). The subcutaneous immunization of C57BL/6N mice with recombinant Pgp3 protein induced a significantly lower organism burden in the lungs following *C. muridarum* airway infection (16). BALB/c mice intranasally immunized with recombinant Pgp3 protein purified from chlamydial serovar D exhibited cross-serovar protection against *C. muridarum* genital tract infection (17). Furthermore, immunization with *Chlamydia psitaci* CPSIT_P7, homologous of *C. trachomatis* Pgp3 with 70% protein sequence identity, provided partial protection against *C. psitaci* lung infection in BALB/c mice (18). Therefore, Pgp3 showed stable protective efficacy against both *Chlamydia* strains and in different mouse models, suggesting the potential value of Pgp3 in clinical vaccine development. However, whether the trimeric conformation of Pgp3 is necessary for its protective efficacy has not been investigated so far, and the answer to this question will guide the design of Pgp3-based multi-epitope peptide and multi-subunit chlamydial vaccines.

In the present study, we tested the efficacy of vaccination with *the C. trachomatis* Pgp3 trimer in comparison with a heat-denatured Pgp3 monomer with a Th1-polarizing adjuvant CpG in a *C. muridarum* murine genital tract model. Both Pgp3 trimer and monomer immunization induced corresponding specific antibody production, but only the trimer-induced antibody recognized endogenous Pgp3, and trimer-immunized mouse splenocytes showed the highest IFN-γ production upon restimulation with chlamydial EB or native Pgp3 *in vitro*. Importantly, only Pgp3 trimer-immunized mice showed shortened lower genital tract chlamydial shedding and decreased upper genital tract pathology, which emphasizes the importance of maintaining the Pgp3 conformation for its protective efficacy. These observations provide important and novel information for the development of chlamydial vaccines.

## Materials and methods

### Standard strain of *Chlamydia*

*C. trachomatis* strain D/UW-3/CX and *C. muridarum* strain Nigg were grown on confluent HeLa 229 cells (ATCC, CCL-2.1), and the EBs were purified from the infected cells by Renografin gradients as described previously (19). After washing twice with phosphate-buffered saline, the EBs were suspended in sucrose-phosphate-glutamate buffer and stored at −80°C.

### Preparation of Pgp3 trimer and monomer

The recombinant plasmid pGEX6p/pgp3 was transformed into *Escherichia coli* XL-1 Blue to express GST-tagged *C. trachomatis* strain D/UW-3/CX protein Pgp3, as described previously (17). GST-Pgp3 was purified using glutathione-conjugated agarose beads and cleaved using a precision protease (Amersham Pharmacia Biotech, Inc., Piscataway, NJ, USA) to remove the GST tag. To rule out endotoxin contamination, the purified Pgp3 protein was treated with the ToxinEraser™ Endotoxin Removal Kit (GenScript, Piscataway, NJ, USA), and the endotoxin level was measured using Limulus Amebocyte Lysate (Chinese Horseshoe Crab Reagent Manufactory, Ltd., Xiamen, China). The endotoxin was found to be less than 0.05 endotoxin units (EU)/ml, which could not to lead to non-specific activation of immune cells. Heat-denatured or linearized monomer Pgp3 antigen was prepared by boiling the native endotoxin-free Pgp3 protein for 1, 5, or 10 min. The quality and molecular weight of both the native Pgp3 protein (trimer) and monomer were assessed using 12% native gel electrophoresis and Coomassie blue staining.

### Mice and immunization

Of note, 5-6-week-old special pathogen-free female BALB/c mice purchased from Hunan SJA Laboratory Animal Co., Ltd. were used in our experiment. In total, 45 mice were obtained and divided into three groups of 15 mice each. Two groups of mice were immunized intranasally with purified Pgp3 trimer or monomer protein at a dose of 30 μg mixed with 10 μg of CpG adjuvant (Coralville, IA, USA). The third group was immunized with CpG adjuvant alone and was used as a control. All mice were immunized three times on three different days (days 0, 20, and 30). Serum samples from five mice/group were collected 14 days after the third immunization to analyze antibody production. To determine whether the antiserum from each group of mice contained antibodies that could recognize Pgp3 protein in a conformation-dependent manner, both recombinant and endogenous Pgp3 proteins were used as antigens to react with these mouse sera by western blot or immunofluorescence assay. Twenty days after the third immunization, five mice/group were sacrificed for splenocyte cytokine detection. All animal procedures complied with the guidelines of the Animal Welfare and Ethics Committee of the University of South China.

### Native gel-western blot assay

The 12% native gel used in this experiment was prepared according to the manufacturer’s instructions (Bio-Rad, Hercules, CA, USA). The protein loading buffer used for the native gel (Sigma-Aldrich, St. Louis, MO, USA) was the same as that used for the denaturing gel but without sodium dodecyl sulfate (SDS). Of note, 2-µg purified recombinant Pgp3 protein or heat-denatured protein was solubilized in the native gel protein loading buffer and loaded into gel wells without boiling. After electrophoresis, the proteins in the gels were transferred onto a PVDF membrane (Sangon Biotech Co., Ltd., Shanghai, China) for antibody detection. A pool of sera collected from Pgp3 trimer-, Pgp3 monomer-, or CpG alone-immunized mice was used as the primary antibody, and HRP-conjugated goat anti-mouse IgG was used as the secondary antibody. Protein binding was visualized using an ECL luminescence reagent (Sangon Biotech Co., Ltd.).

### Immunofluorescence assay

HeLa 229 monolayers cultured on coverslips were infected with *C. muridarum* strain Nigg and processed for antibody staining as described previously (19, 20). Infected cells were fixed with 2% paraformaldehyde for 30 min and permeabilized with 1% saponin (Sigma-Aldrich) for 45 min at room temperature. After blocking, Hoechst staining solution (blue; Sangon Biotech Co., Ltd.) was used to visualize DNA. A genus-specific rabbit antibody against chlamydial EBs and Cy2 (green; Jackson ImmunoResearch Laboratories, Inc., West Grove, PA, USA)-labeled goat anti-rabbit IgG secondary antibody were used to visualize the chlamydial inclusions. Antisera collected from each immunized mouse group and Cy3 (red; Jackson ImmunoResearch Laboratories, Inc.)-conjugated goat anti-mouse IgG secondary antibody were used to visualize the Pgp3 antigen. All images were obtained using an AX-70 fluorescence microscope (Olympus, Melville, NY, USA).

### Detection of IgG and IgG subclasses

Ultraviolet-inactivated *C. muridarum* EBs were used as antigens to monitor chlamydia-specific IgG and its subclasses in mouse antisera by enzyme-linked immunosorbent assay (ELISA). Briefly, 96-well plates (Thermo Labsystems, Franklin, MA, USA) were coated with 1 µg/well of EB and kept overnight at 4°C. After blocking with 2.5% milk, serially diluted antisera from each mouse were added, and the plates were incubated at 37°C for 1.5 h. Then, HRP-conjugated goat anti-mouse IgG (H+L), IgG1, IgG2a, IgG2b, or IgG3 secondary antibody and ABTS (Sigma-Aldrich) substrate were added for chlamydia-specific IgG and subclass detection. Absorbance (OD) was measured at 405 nm using a microplate reader (BioTek Instruments, Winooski, VT, USA).

### Detection of antigen-specific IFN-γ production

Antigen-specific IFN-γ production was evaluated using ELISA as described previously (21, 22). Splenocyte single-cell suspensions prepared from each mouse (5 mice/group) 20 days after the third immunization were inoculated (1 × 10^6^ cells/well) into 96-well culture plates with no treatment (medium), or 1μg per well of an unrelated protein fetal bovine serum (BSA), or UV-inactivated *C. muridarum* EBs at 1 × 10^6^ IFUs/well, or 1μg per well of recombinant Pgp3 (native Pgp3), or heat-denatured Pgp3. Then, 72 h after stimulation, the supernatants were collected and evaluated using an IFN-γ ELISA kit (Sangon Biotech Co., Ltd.).

### Mouse urogenital tract infection and monitoring of chlamydial shedding

Twenty-five days after the final immunization, 2.5 mg of Depo-Provera/mouse was administered subcutaneously to synchronize mice estrous cycles. Five days later, each mouse was challenged intravaginally (i.vag.) with 2 × 10^4^ inclusion forming units (IFUs) of *C. muridarum*. Vaginal swabs were taken every 4 days until 28 days after infection to monitor chlamydial shedding in the lower genital tract of the mice. Each swab sample was diluted appropriately and used to infect HeLa 229 monolayers as described previously (17, 22). After 24 h of incubation, inclusion bodies in the culture were visualized by immunofluorescence assay and counted under a microscope. The total number of IFUs per swab was calculated and converted into log10, and log10 IFUs were used to report the mean ± SD per group of mice at each time point.

### Pathological evaluation

Sixty days post-challenge, all mice were sacrificed for pathological evaluation. First, the entire genital tract, including the vagina, cervix, uterine horn, oviduct, and ovary, was checked for swelling, inflammation, and adhesion changes *in situ*. Under the naked eye, the hydrosalpinx of the oviduct was obvious, and the severity was scored as reported previously (17, 23). Briefly, the ovary of each mouse was used as a size reference: 0, no hydrosalpinx; 1, hydrosalpinx suspicious, not sure; 2, hydrosalpinx is clear and its size is smaller than that of the ovary; 3, similar size to that of the ovary; and 4, larger than the ovary. The entire genital tract was isolated for H&E staining and assessed by a pathologist. Under a microscope, changes in the uterine horn and oviduct were obvious. The severity was scored separately using the double-blind method, and the scoring criteria were as follows. Dilation of the uterine horn or oviduct: 0, no significant dilation; 1, mild dilation of a single cross-section; 2, one to three dilated cross-sections; 3, more than three dilated cross-sections; and 4, confluent pronounced dilation. Inflammatory cell infiltration (at the chronic stage of infection, the inflammatory cells that infiltrate are mainly mononuclear cells): 0, no significant infiltration; 1, infiltration at a single focus; 2, infiltration at two to four foci; 3, infiltration at more than four foci; and 4, confluent infiltration. The scores assigned to each mouse were used to report the mean ± SD of each group of mice.

### Statistical analysis

ANOVA test was used to analyze data from three groups. A two-tailed Student’s *t*-test *via* GraphPad Prism 6.0 software (GraphPad Software, Inc., La Jolla, CA) was used to compare means between two groups. A Wilcoxon rank sum test (SPSS 20.0) was used to analyze differences in the speed of clearance and a Kruskal-Wallis test was used to analyzed ranked pathology scores. A chi-square test (Microsoft Excel) was performed to compare the incidence rates between two groups. * *p* < 0.05 and ** *p* < 0.01 indicated statistical significance.

## Results

### Generation of Pgp3 trimer and denatured monomer

**Figure 1:**
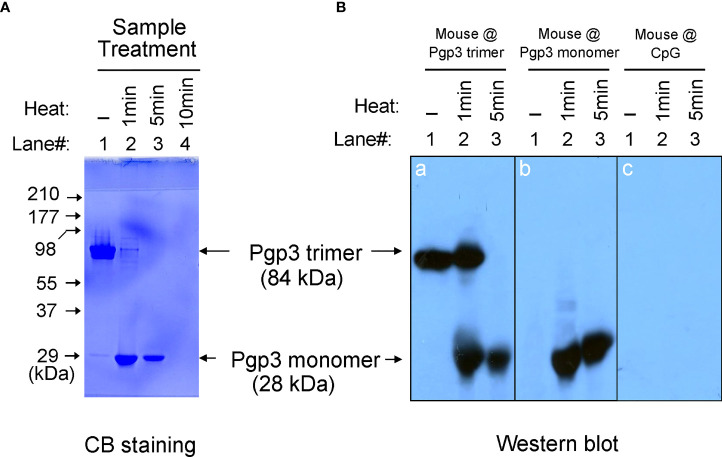
Pgp3 proteins used in this study and their immunogenic analysis. **(A)** Untreated Pgp3 protein and Pgp3 protein boiled for 1, 5, or 10 min were loaded in a 12% native gel and stained with Coomassie blue dye. **(B)** Untreated Pgp3 protein and Pgp3 protein boiled for 1 or 5 min were loaded in gels for western blot detection with sera immunized with Pgp3 trimer (panel **a**), Pgp3 monomer (panel **b**), or CpG alone (panel **c**).

It has been previously reported that recombinant Pgp3 protein purified from a prokaryotic expression system forms a stable trimer (13). In the present study, Pgp3 was expressed in *E. coli* as a GST-tagged fusion protein and purified using glutathione-conjugated agarose beads. The GST tag was removed using a precision protease. The Pgp3 protein migrated to ~84 kDa in the native gel, corresponding to the molecular weight of trimeric Pgp3 (**Figure 1A**). To generate the Pgp3 monomer, aliquots of recombinant Pgp3 protein were boiled for 1, 5, or 10 min. As expected, heating at 100°C gradually changed Pgp3 trimers into monomers. After 5 min of boiling, all proteins migrated to ~28 kDa in the native gel, corresponding to the molecular weight of monomeric Pgp3. After 10 min of boiling, all proteins were degraded (**Figure 1A**). Therefore, untreated native Pgp3 trimer and 5-min heat-denatured Pgp3 monomer were used for the following vaccination experiment.

### Both Pgp3 trimer and monomer immunization induce specific antibody responses

To determine whether the conformation of the Pgp3 protein affects specific antibody production, sera collected from five mice of each group were evaluated using a native gel-western blot assay. Recombinant Pgp3 with or without 1- or 5-min heat treatment served as antigens, and immunization with the Pgp3 trimer (**Figure 1B** **, panel a**) or monomer (**Figure 1B** **, panel b**) but not CpG alone (**Figure 1B** **, panel c**) induced the production of Pgp3-specific antibodies. Interestingly, trimer-immunized mouse sera reacted with both trimeric and monomeric Pgp3, but monomer-immunized mouse sera only recognized the monomeric Ppg3. These results suggest that the Pgp3 trimer contains both conformational and linear B cell epitopes, and the linear ones may be inside the molecule, whereas the 5-min heat-denatured monomer loses conformational epitopes but exposes linear epitopes.

**Figure 2:**
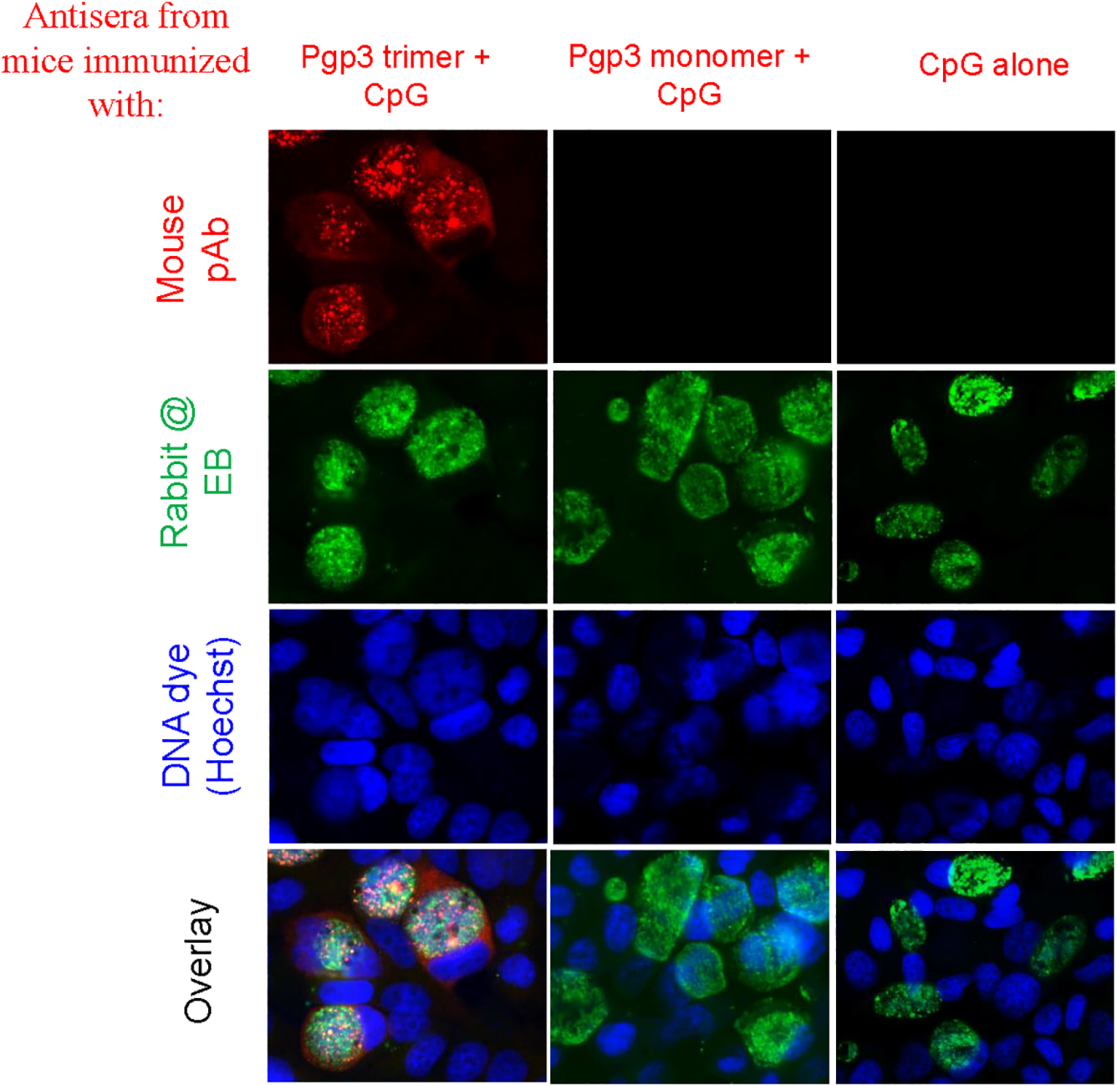
Detection of antibodies against endogenous Pgp3 in *C. muridarum*-infected cell samples. Sera collected from each group were used to detect endogenous Pgp3 by an immunofluorescence assay. *C. muridarum*-infected cells were costained with mice antisera (red) as indicated at the top of the figure, rabbit anti-chlamydial EB polyclone antibody (green), and DNA dye (blue). Only Pgp3 trimer-immunized mice produced antibodies that recognized endogenous Pgp3.

**Figure 3:**
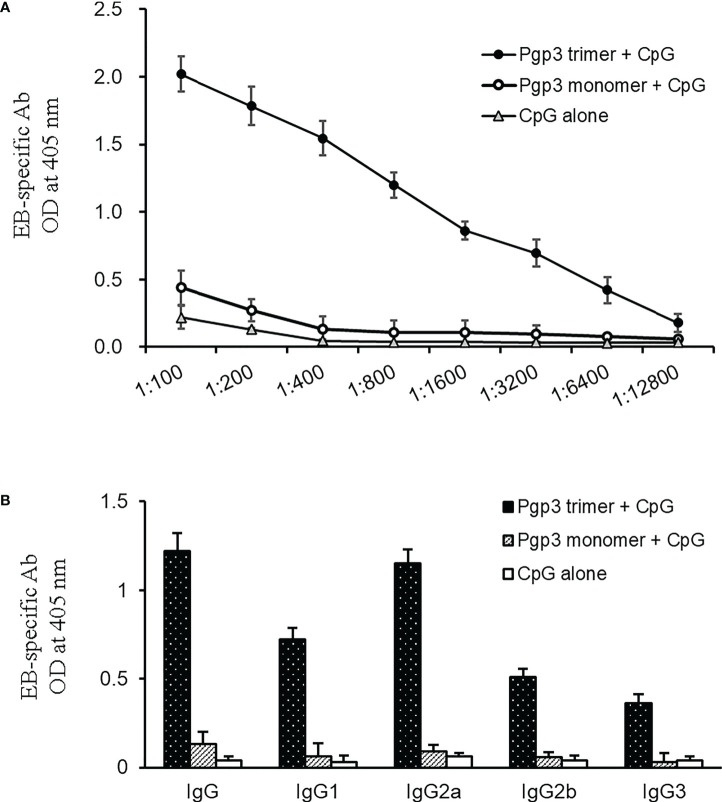
Detection of (*C*) *muridarum* EB-specific antibody responses by ELISA. **(A)** (*C*) *muridarum* EB was coated onto 96-well plates as antigen, and the serially diluted mice sera collected from each group were applied to the microplates. Antibody binding was detected, and the results are shown as OD values. **(B)** Serum from each mouse after 1:800 dilution was made to react with coated EB antigen to analyze IgG subclasses by ELISA. Results are representative of three independent experiments.

To determine whether Pgp3 monomer-induced specific antibodies were able to recognize the endogenous Pgp3 protein, HeLa monolayers infected with *C. muridarum* were processed for mouse antisera staining using an immunofluorescence assay. As shown in **Figure 2**, endogenous Pgp3 was stained only in Pgp3 trimer-immunized mouse sera. *C. muridarum* EB-specific antibody titers were further quantitated using ELISA, which showed that only Pgp3 trimer-immunized mice developed high titers of anti-EB total IgG, IgG1, IgG2a, IgG2b, and IgG3 (**Figure 3**). However, Pgp3 monomer- and CpG alone-immunized mice displayed minimal levels of anti-EB antibodies. Collectively, these observations suggest that both Pgp3 trimers and monomers are immunogenic but that they induce different specific antibodies. Only sera from Pgp3 trimer-immunized mice recognized endogenous Pgp3, which is located in the host cell cytosol, in the inclusion lumen, and on the chlamydial EB surface.

### Pgp3 immunization induces INF-γ production

**Figure 4:**
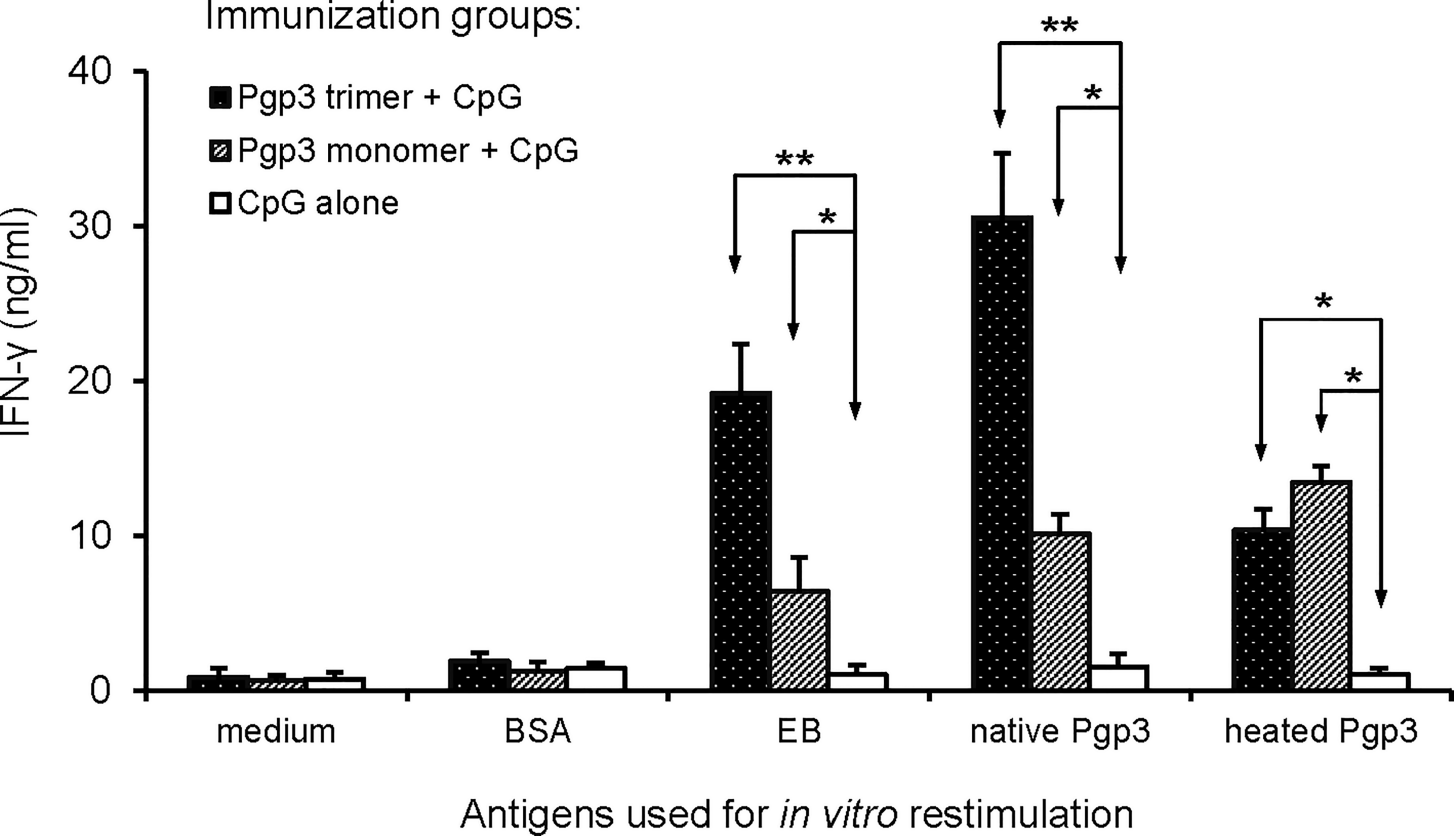
Induction of antigen-specific IFN-γ production following vaccination. Twenty days after the last immunization, splenocytes harvested from each group (5 mice/group) were restimulated *in vitro* with the following antigens as listed along the *X-axis*: medium alone, BSA at the concentration of 10ug/ml (1μg per well), UV-inactivated *C. muridarum* EBs at 1 × 10^6^ IFUs/well, native Pgp3 and heated Pgp3 at 10ug/ml. IFN-γ production was evaluated 72 h after stimulation using an indirect ELISA. Results are expressed as ng/ml, as listed along the *Y-axis* (mean ± standard deviation). Results are representative of three independent experiments (* *p* < 0.05, ** *p* < 0.01).

Since the Th1 signature cytokine INF-γ is critical for the clearance of chlamydia infection, INF-γ levels have been an important indicator for evaluating the protective efficacy of a vaccine candidate antigen. In this study, splenocytes prepared from each mouse (5 mice/group) were inoculated into plates and stimulated with medium, unrelated antigen BSA, EB, native Pgp3, or heated Pgp3. IFN-γ production was evaluated using ELISA **(** **Figure 4**). Compared with the CpG alone-immunized group, IFN-γ secretion in both Pgp3-immunized groups was significantly higher. It is worth noting that when the splenocytes were restimulated with EB or native Pgp3 *in vitro*, the production of IFN-γ was much more robust in the Pgp3 trimer-immunized group than in the Pgp3 monomer-immunized group.

### Kinetics of live chlamydial organism shedding in vaccinated mice after challenge

**Figure 5:**
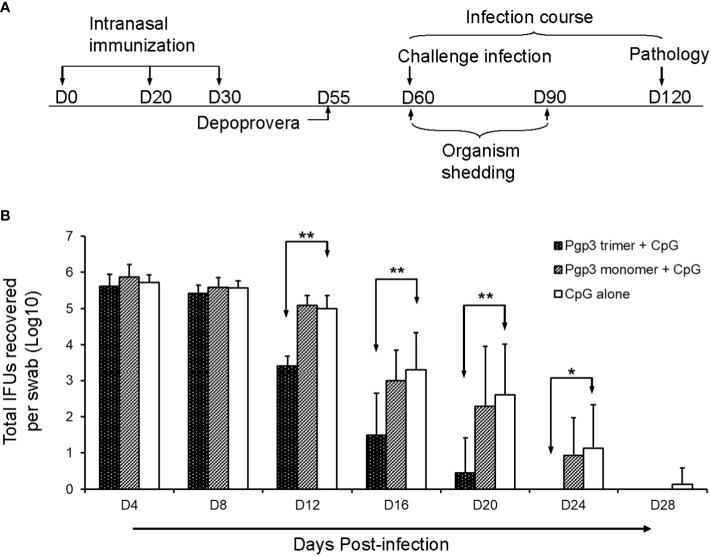
Live chlamydial organism shedding from mice vaginas following challenge. **(A)** For the experiment, 5-6-week-old BALB/c mice were immunized intranasally with Pgp3 trimer + CpG, Pgp3 monomer + CpG, or CpG alone on days 0, 20, and 30. In total, five mice/group were sacrificed before the challenge to monitor specific humoral and cellular immune responses. The remaining mice (n = 10) were challenged intravaginally with (*C*) *muridarum*, and vaginal live chlamydial organism shedding was monitored every 4 days until 28 days after infection. Day 60 post-challenge, all mice were sacrificed for pathological evaluation. **(B)** Vaginal swabs were collected for measuring IFU numbers, and each swab IFU number was converted into log_10_. The log_10_ IFUs were compared between the three groups of mice at each time point using the ANOVA Test and a Wilcoxon rank sum test was used to further analyze the speed of clearance. Significant differences were observed between the Pgp3 trimer versus CpG alone groups. ** *p* < 0.01, * *p* < 0.05, analyzed by ANOVA and a Wilcoxon rank sum test.

To evaluate whether the Pgp3 conformational structure is expendable for the induction of protective immunity, 60 days after the first immunization, all mice were challenged i.vag. with 2 × 10^4^ IFUs of *C. muridarum*. Vaginal chlamydial shedding was monitored every 4 days until 28 days after the challenge. While Pgp3 trimer-immunized mice exhibited a significant reduction in live chlamydial organism shedding on days 12, 16, 20, and 24 compared with CpG alone-immunized mice, there was no significant reduction observed in Pgp3 monomer-immunized mice (**Figure 5**). Moreover, 80% of the Pgp3 trimer-immunized mice stopped chlamydial shedding by day 20 post-infection, and all mice showed resolution of infection by day 24. In comparison, 70% or more mice in the Pgp3 monomer- and CpG alone-immunized groups exhibited positive chlamydial shedding by day 20, with 50% mice in these groups continuing shedding by day 24 (**Table 1**). These results indicate that only Pgp3 trimer immunization accelerated chlamydial clearance from the lower genital tract of mice.

**Table 1:** Percentage of positive chlamydial shedding in diverse immunization groups.

Immunization group	No. of mice	No. of mice shedding chlamydia from the vagina (%)						
		Days after intravaginal challenge						
		D4	D8	D12	D16	D20	D24	D28
Pgp3 trimer + CpG	10	10 (100)	10 (100)	10 (100)	7 (70)	2 (20) ^**^	0 (0) ^**^	0 (0)
Pgp3 monomer + CpG	10	10 (100)	10 (100)	10 (100)	10 (100)	7 (70)	5 (50)	0 (0)
CpG alone	10	10 (100)	10 (100)	10 (100)	10 (100)	8 (80)	5 (50)	1 (10)

** p < 0.01 vs. CpG alone analyzed by ANOVA and a chi-square test.

### Pgp3 trimer vaccination alleviates mice upper genital tract pathology

**Figure 6:**
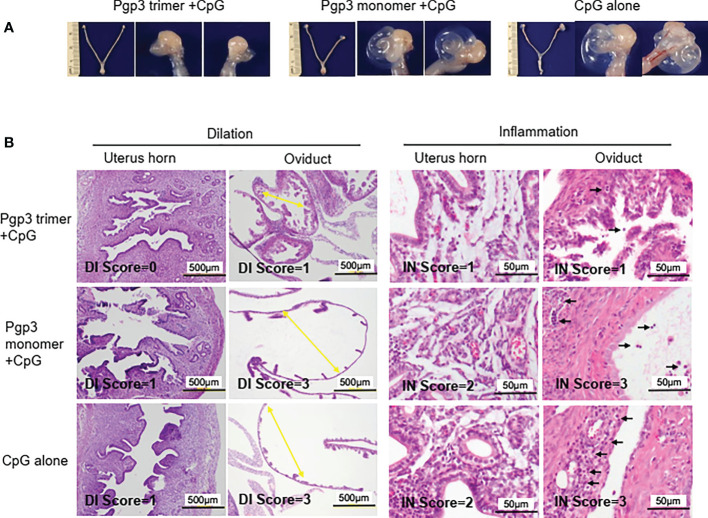
Pgp3 trimer immunization reduced *C. muridarum*-induced upper genital tract pathology. Sixty days after *C. muridarum* infection, mice were sacrificed for pathology observation. **(A)** The urogenital tract tissues from mice immunized with Pgp3 trimer + CpG, Pgp3 monomer + CpG or CpG alone were examined at the level of gross appearance. Representative images of the intact urogenital tract tissues and corresponding amplified oviduct/ovary from both left and right side were presented. **(B)** The urogenital tract tissues were sectioned for microscopic observation of histological pathologies. Representative images from each group containing the uterine horn or oviduct are shown. DI, luminal dilation (yellow arrow); IN, inflammatory cell infiltration (black arrow).

On day 60 after the challenge, all mice were sacrificed for pathological evaluation. We found that the reproductive tract did not adhere to the surrounding organs or tissues. No obvious pathological changes in the vagina, cervix, uterine horn, and ovary were observed under the naked eye, but the severity of oviduct blockage (hydrosalpinx) varied greatly among the three groups of mice. As shown in **Table 2**, 90% (60% bilateral) of the Pgp3 monomer- and 80% (70% bilateral) of the CpG alone-immunized mice developed hydrosalpinx. In comparison, the incidence of bilateral hydrosalpinx was significantly lower (20%) in the Pgp3 trimer-immunized mice. In total, 15 of 20 oviducts (75%) from the Pgp3 monomer- or CpG alone-immunized mice developed hydrosalpinx, which was significantly higher than that from the Pgp3 trimer-immunized mice (7 of 20; 35%). Images of the intact urogenital tract tissues and corresponding amplified oviduct/ovary from both left and right side are shown (**Figure 6A**, **Supplementary Figure 1**). We used a scoring system for semi-quantitatively assessing the severity of hydrosalpinx in each oviduct. Multi sample Kruskal Wallis H test was performed to compare hydrosalpinx severity scores among three groups, which indicates statistical difference (H=12.503, p=0.002). This observation suggests that Pgp3 trimer + CpG immunization reduce the progression of hydrosalpinx. After gross appearance assessment, we further evaluated the severity of inflammation microscopically using histology sections (**Figure 6B**). Inflammatory infiltrates and luminal dilatation were significantly lower in oviduct tissues of mice treated with Pgp3 trimer + CpG. These results demonstrate that the conformation of Pgp3 affects its protective efficacy.

**Table 2:** Hydrosalpinx data in diverse immunization groups.

Immunization group	Number of mice with hydrosalpinx/total mice (%) *^a^*	Number of mice with bilateral hydrosalpinx/total mice (%) *^a^*	Number of oviducts with hydrosalpinx/total oviducts (%) *^a^*	Kruskal-Wallis test *^b^*	
				H	*P value*
Pgp3 trimer + CpG	5/10 (50)	2/10 (20) * *^p < 0.05^*	7/20 (35) * *^p<0.05^*		
Pgp3 monomer + CpG	9/10 (90)	6/10 (60)	15/20 (75)	12.503	0.002
CpG alone	8/10 (80)	7/10 (70)	15/20 (75)		

^a^ vs. CpG alone analyzed by ANOVA and a chi-square test. * *p* < 0.05.

^b^ Kruskal-Wallis test was used to compare hydrosalpinx severity scores among three groups, which indicates statistical difference (p=0.002).

## Discussion

Previously, we reported that immunization with recombinant Pgp3 plus CpG adjuvant induced cross-serovar protection against *C. muridarum* genital tract infections (17). The native conformation of Pgp3 is homotrimeric when it is endogenously expressed in infected cells or purified from an *E. coli* expression system. Treatment with SDS, urea, boiling, or various combinations of denaturing methods converts portions of or all Pgp3 trimers into monomers (13). To assess whether the conformational structure is expendable for Pgp3 to provide anti-chlamydial immunity, we immunized mice with recombinant Pgp3 trimers or heat-denatured monomers. We found that both immunizations induced specific antibody production; however, only trimer-induced antisera recognized endogenous Pgp3, and trimers induced the highest EB-specific IFN-γ production. Importantly, only Pgp3 trimer immunization significantly shortened lower genital tract chlamydial shedding and decreased upper genital tract pathology, and the protective efficacy of the Pgp3 trimer was better than that of the Pgp3 monomer.

To assess whether protein-induced immune protection is related to conformation, the protein conformation should be damaged, but the primary structure should be preserved (24). Boiling with water denatures most proteins *via* the destruction of hydrogen bonds, resulting in the loss of secondary and higher structures. However, moderately high-temperature treatment may result in proteins that retain the primary structure, but too high-temperature treatment or too long treatment time may lead to protein aggregation and immunogenicity disappearance (25). Based on our previous studies (13), recombinant Pgp3 purified from a bacterial expression system is a stable trimer and a mouse polyclonal antibody raised with a purified Pgp3 protein recognize both native and denatured Pgp3. To monitor the oligomeric status of Pgp3, the purified Pgp3 left untreated or treated with boiling were analyzed using a 12% native gel plus a Western blot assay (**Figure 1**). Note that the untreated Pgp3 migrated at the ∼84 kDa position (corresponding to the molecular mass of trimeric Pgp3) whereas the Pgp3 treated with 5 min of boiling migrated at the ∼28 kDa position (corresponding to the monomeric form of Pgp3). Moreover, after 5 min boiling, the mouse anti-Pgp3 polyclonal antibody known to recognize both native and denatured Pgp3 could not detect any Pgp3 trimer band in Western blot assay which demonstrated that heating for 5 min converted all Pgp3 trimers into monomers. However, it was unclear whether this heat-denatured monomer was immunogenic. To confirm this, we assessed the specific humoral and cytokine recall responses to Ppg3 monomer immunization. We found that monomer-induced mouse antisera recognized the Pgp3 monomer in the western blot assay but CpG alone-immunized mouse sera could not, and the splenocyte IFN-γ production level in monomer-immunized mice was significantly higher than that in control mice upon heated-Pgp3 restimulation *in vitro*. Chaganty et al. also showed that boiling CPAF for 5 min resulted in the loss of enzymatic activity (dependent on tertiary conformation), but the linear epitopes were unaltered (24). In our study, high-temperature treatment destroyed the conformational structure but preserved the immunogenicity, which is consistent with Chaganty et al.’s findings, despite the use of different protein immunogens.

The Pgp3 monomer has good immunogenicity, but it induces humoral and cytokine immune responses that differ significantly from those induced by Pgp3 trimers, which may explain the differences in their immune protective efficacy. Mouse antisera collected after Pgp3 monomer immunization could not react with trimerized Pgp3 in the western blot assay and could not recognize endogenous Pgp3 in chlamydia-infected cells, suggesting that Pgp3 monomer-induced antibodies may not play a role during host chlamydial infection, which was consistent with Kari’s finding that the Pgp3 antibody correlated with chlamydial infection eradication and that an efficacious vaccine requires Ppg3 and other chlamydial immunodominant antigens delivered in their native conformation (26). However, the Tímea Mosolygó’ chlamydial airway infection mouse model showed that even antisera obtained against native Pgp3 could not reduce the amount of chlamydia in the lungs of mice (16). Chaganty et al. reported that vaccination with heat-denatured CPAF induced very low levels of CPAF-specific antibody production but provided comparable protection to native CPAF, indicating that the role of specific antibodies against chlamydial infection is negligible (24).

Although the role of specific humoral immune responses in controlling chlamydial infection is controversial, the role of Th1 cells and their signature cytokine IFN-γ is well defined (27–29). The Pgp3 monomer and trimer may differ in conformational structure, but the primary amino acid sequence and linear epitopes are identical, and Th1 cell activation relies on antigen-presenting cells (APC) presenting linear epitopes. Thus, Pgp3 monomer- and trimer-induced Th1 cytokine IFN-γ production was expected to be identical, similar to how heat-denatured CPAF induced comparable levels of IFN-γ production to native CPAF. Unfortunately, upon EB or native Pgp3 restimulation *in vitro*, the splenocyte IFN-γ levels in the monomer group were significantly lower than those in the trimer group. We suggest three possible reasons for this. First, Pgp3 trimers are more complex than monomers and may provide more epitopes to stimulate the immune system. Second, the C-terminal domain of the Pgp3 trimer is the closest bacterial homolog to human and rodent TNF family members (10), which may play a role in host cell binding and may facilitate the APC uptake of Pgp3 antigen and presentation to Th1 cells. Third, the Pgp3 trimer can bind to and form a stable complex with cathelicidin peptide LL-37 to enhance proinflammatory activity on myeloid cells (30), which may stimulate APC to express costimulation molecules and improve antigen presentation. It will be interesting to further study the role of the pooled, overlapping peptides of the Pgp3 protein and its induced Abs and cytokine responses to examine whether these responses were dependent on conformation or APC activation status.

Immunization with the Pgp3 monomer does not protect against chlamydial urogenital tract challenge. Pgp3 trimer-immunized mice showed reduced chlamydial shedding on day 12 and enhanced clearance by day 20, but Pgp3 monomer-immunized mice showed a similar shedding time course and clearance pattern to CpG alone-immunized mice. Moreover, only Pgp3 trimer vaccination significantly decreased upper genital tract pathology. The present study used a well-established chlamydial infection animal model, and the protective efficacy of the Pgp3 trimer is consistent with previous observations (17), which confirmed that conformational structure and conformational epitopes are indispensable for Ppg3 vaccine protection. This finding has important implications for the design of Pgp3-based chlamydial vaccines for human use. First, SDS and other detergents should not be used in Pgp3 preparation, either from a bacterial expression system or EB organism outer membrane complex, since the Pgp3 trimer contains SDS-accessible cysteine residues (13). Second, boiling Pgp3 for 1 min caused a large amount of trimer to be converted to monomer; hence, high temperatures cannot be used during vaccine preparation. Thus, low-temperature storage and transportation are necessary. Third, Pgp3 must be in the native conformation to induce high IFN-γ production, suggesting that a multi-epitope peptide vaccine should be designed with a small fragment of Pgp3 that includes both conformational and linear epitopes. Finally, since the chlamydial multi-subunit vaccine usually offers better protection than the mono-component subunit vaccine and the Pgp3 conformation is likely maintained by the C-terminal 75% amino acid sequence (10, 31–33), it would be interesting to test whether combined subunit candidates, such as MOMP-Pgp3_C_ or CPAF-Pgp3_C_, form trimers and provide better protection than the corresponding single-subunit vaccines.

In summary, the results of this study, in addition to those from our previous studies, indicate that Pgp3 is a promising vaccine candidate, and its protective efficacy against *Chlamydia* urogenital tract infection is highly dependent on the native conformation. Our findings provide novel and important information for the development of Pgp3 as a clinical chlamydial vaccine.

## Data availability statement

The original contributions presented in the study are included in the article/**Supplementary Material**. Further inquiries can be directed to the corresponding author.

## Ethics statement

The animal study was reviewed and approved by the Animal Use and Ethics Committee of the University of South China.

## Author contributions

Conceived and designed the experiments: CL. Performed the experiments: BP, SZ, YH, and QL. Analyzed the data: CL, BP, CW, ZL, CY, and AL. Wrote the paper: BP and CL. All authors contributed to the article and approved the submitted version.

## Funding

This work was supported by the National Natural Science Foundation of China (grant number 81471969), Natural Science Foundation of Hunan Province (grant number 2022JJ30505), Innovation Platform Open Fund Project of Hunan Provincial Education Department (grant number 20K108), and Hunan Province Students’ Innovation and Entrepreneurship Training Program (Xiang Jiao Tong [2022] number 174, grant number S202210555244).

## Acknowledgments

We extend our sincere thanks to Dr. Guangming Zhong from the Department of Microbiology, Immunology, and Molecular Genetics, University of Texas Health Science Center at San Antonio, San Antonio, Texas, USA, who provided the chlamydial organisms and several good suggestions for this study. We would like to thank Editage (www.editage.com) for English language editing.

## Conflict of interest

The authors declare that the research was conducted in the absence of any commercial or financial relationships that could be construed as a potential conflict of interest.

## Publisher’s note

All claims expressed in this article are solely those of the authors and do not necessarily represent those of their affiliated organizations, or those of the publisher, the editors and the reviewers. Any product that may be evaluated in this article, or claim that may be made by its manufacturer, is not guaranteed or endorsed by the publisher.

## References

[1] Brunham RC. Problems with understanding *Chlamydia trachomatis* immunology. J Infect Dis (2022) 225:2043–9. doi: 10.1093/infdis/jiab610 34919679

[2] Murray SM McKay PF. *Chlamydia trachomatis*: cell biology, immunology and vaccination. Vaccine (2021) 39:2965–75. doi: 10.1016/j.vaccine.2021.03.043 33771390

[3] Kreisel KM Spicknall IH Gargano JW Lewis FMT Lewis RM Markowitz LE. Sexually transmitted infections among us women and men: prevalence and incidence estimates, 2018. Sex Transm Dis (2021) 48:208–14. doi: 10.1097/OLQ.0000000000001355 PMC10245608 33492089

[4] Golden MR Whittington WL Handsfield HH Hughes JP Stamm WE Hogben M. Effect of expedited treatment of sex partners on recurrent or persistent gonorrhea or chlamydial infection. N Engl J Med (2005) 352:676–85. doi: 10.1056/NEJMoa041681 15716561

[5] Geisler WM. Duration of untreated, uncomplicated *Chlamydia trachomatis* genital infection and factors associated with chlamydia resolution: a review of human studies. J Infect Dis (2010) 201:S104–13. doi: 10.1086/652402 20470048

[6] Adachi KN Nielsen-Saines K Klausner JD. *Chlamydia trachomatis* screening and treatment in pregnancy to reduce adverse pregnancy and neonatal outcomes: a review. Front Public Health (2021) 9:531073. doi: 10.3389/fpubh.2021.531073 34178906 PMC8222807

[7] Chen J Zhang H Zhou Z Yang Z Ding Y Zhou Z. Chlamydial induction of hydrosalpinx in 11 strains of mice reveals multiple host mechanisms for preventing upper genital tract pathology. PloS One (2014) 9:e95076. doi: 10.1371/journal.pone.0095076 24736397 PMC3988139

[8] Liu Y Huang Y Yang Z Sun Y Gong S Hou S. Plasmid-encoded Pgp3 is a major virulence factor for *Chlamydia muridarum* to induce hydrosalpinx in mice. Infect Immun (2014) 82:5327–35. doi: 10.1128/IAI.02576-14 PMC4249284 25287930

[9] Shao L Zhang T Melero J Huang Y Liu Y Liu Q. The genital tract virulence factor Pgp3 is essential for *Chlamydia muridarum* colonization in the gastrointestinal tract. Infect Immun (2018) 86:e00429–17. doi: 10.1128/IAI.00429-17 PMC5736818 29038127

[10] Galaleldeen A Taylor AB Chen D Schuermann JP Holloway SP Hou S. Structure of the *Chlamydia trachomatis* immunodominant antigen Pgp3. J Biol Chem (2013) 288:22068–79. doi: 10.1074/jbc.M113.475012 PMC3724661 23703617

[11] Huang Y Sun Y Qin T Liu Y. The structural integrity of plasmid-encoded Pgp3 is essential for induction of hydrosalpinx by chlamydia muridarum. Front Cell Infect Microbiol (2019) 9:13. doi: 10.3389/fcimb.2019.00013 30805313 PMC6370636

[12] Li Z Chen D Zhong Y Wang S Zhong G. The chlamydial plasmid-encoded protein Pgp3 is secreted into the cytosol of chlamydia-infected cells. Infect Immun (2008) 76:3415–28. doi: 10.1128/IAI.01377-07 PMC2493211 18474640

[13] Chen D Lei L Lu C Galaleldeen A Hart PJ Zhong G. Characterization of Pgp3, a *Chlamydia trachomatis* plasmid-encoded immunodominant antigen. J Bacteriol (2010) 192:6017–24. doi: 10.1128/JB.00847-10 PMC2976438 20851898

[14] Wang J Zhang Y Lu C Lei L Yu P Zhong G. A genome-wide profiling of the humoral immune response to *Chlamydia trachomatis* infection reveals vaccine candidate antigens expressed in humans. J Immunol (2010) 185:1670–80. doi: 10.4049/jimmunol.1001240 20581152

[15] Donati M Sambri V Comanducci M Di Leo K Storni E Giacani L. DNA Immunization with Pgp3 gene of *Chlamydia trachomatis* inhibits the spread of chlamydial infection from the lower to the upper genital tract in C3h/Hen mice. Vaccine (2003) 21:1089–93. doi: 10.1016/s0264-410x(02)00631-x 12559784

[16] Mosolygo T Szabo AM Balogh EP Faludi I Virok DP Endresz V. Protection promoted by Pgp3 or Pgp4 against *Chlamydia muridarum* is mediated by Cd4(+) cells in C57bl/6n mice. Vaccine (2014) 32:5228–33. doi: 10.1016/j.vaccine.2014.07.039 25077421

[17] Luan X Peng B Li Z Tang L Chen C Chen L. Vaccination with mip or Pgp3 induces cross-serovar protection against chlamydial genital tract infection in mice. Immunobiology (2019) 224:223–30. doi: 10.1016/j.imbio.2018.11.009 30558842

[18] Tan Y Li Y Zhang Y Yu J Wen Y Wang C. Immunization with *Chlamydia psittaci* plasmid-encoded protein Cpsit_P7 induces partial protective immunity against chlamydia lung infection in mice. Immunol Res (2018) 66:471–9. doi: 10.1007/s12026-018-9018-3 30097797

[19] Lu C Zeng H Li Z Lei L Yeh IT Wu Y. Protective immunity against mouse upper genital tract pathology correlates with high IFNγ but low IL-17 T cell and anti-secretion protein antibody responses induced by replicating chlamydial organisms in the airway. Vaccine (2012) 30:475–85. doi: 10.1016/j.vaccine.2011.10.059 PMC3246108 22079265

[20] Lu C Lei L Peng B Tang L Ding H Gong S. *Chlamydia trachomatis* GlgA is secreted into host cell cytoplasm. PloS One (2013) 8:e68764. doi: 10.1371/journal.pone.0068764 23894341 PMC3722199

[21] Li Z Lu C Peng B Zeng H Zhou Z Wu Y. Induction of protective immunity against *Chlamydia muridarum* intravaginal infection with a chlamydial glycogen phosphorylase. PloS One (2012) 7:e32997. doi: 10.1371/journal.pone.0032997 22427926 PMC3299733

[22] Lu C Peng B Li Z Lei L Li Z Chen L. Induction of protective immunity against *Chlamydia muridarum* intravaginal infection with the chlamydial immunodominant antigen macrophage infectivity potentiator. Microbes Infect (2013) 15:329–38. doi: 10.1016/j.micinf.2013.02.001 PMC4218745 23416214

[23] Peng B Lu C Tang L Yeh IT He Z Wu Y. Enhanced upper genital tract pathologies by blocking Tim-3 and pd-L1 signaling pathways in mice intravaginally infected with chlamydia muridarum. BMC Infect Dis (2011) 11:347. doi: 10.1186/1471-2334-11-347 22168579 PMC3259114

[24] Chaganty BK Murthy AK Evani SJ Li W Guentzel MN Chambers JP. Heat denatured enzymatically inactive recombinant chlamydial protease-like activity factor induces robust protective immunity against genital chlamydial challenge. Vaccine (2010) 28:2323–9. doi: 10.1016/j.vaccine.2009.12.064 PMC2846592 20056182

[25] Chen H Peng B Yang C Xie L Zhong S Sun Z. The role of an enzymatically inactive CPAF mutant vaccination in *Chlamydia muridarum* genital tract infection. Microb Pathog (2021) 160:105137. doi: 10.1016/j.micpath.2021.105137 34390765

[26] Kari L Bakios LE Goheen MM Bess LN Watkins HS Southern TR. Antibody signature of spontaneous clearance of *Chlamydia trachomatis* ocular infection and partial resistance against re-challenge in a nonhuman primate trachoma model. PloS Negl Trop Dis (2013) 7:e2248. doi: 10.1371/journal.pntd.0002248 23738030 PMC3667776

[27] Rixon JA Depew CE McSorley SJ. Th1 cells are dispensable for primary clearance of chlamydia from the female reproductive tract of mice. PloS Pathog (2022) 18:e1010333. doi: 10.1371/journal.ppat.1010333 35196366 PMC8901068

[28] Gondek DC Roan NR Starnbach MN. T Cell responses in the absence of IFN-γ exacerbate uterine infection with chlamydia trachomatis. J Immunol (2009) 183:1313–9. doi: 10.4049/jimmunol.0900295 PMC2723820 19561106

[29] Sahu R Dixit S Verma R Duncan SA Smith L Giambartolomei GH. Encapsulation of recombinant MOMP in extended-releasing PLGA 85: 15 nanoparticles confer protective immunity against a *Chlamydia muridarum* genital challenge and re-challenge. Front Immunol (2021) 12:660932. doi: 10.3389/fimmu.2021.660932 33936096 PMC8081181

[30] Hou S Sun X Dong X Lin H Tang L Xue M. Chlamydial plasmid-encoded virulence factor Pgp3 interacts with human cathelicidin peptide ll-37 to modulate immune response. Microbes Infect (2019) 21:50–5. doi: 10.1016/j.micinf.2018.06.003 PMC6309935 29959096

[31] Boje S Olsen AW Erneholm K Agerholm JS Jungersen G Andersen P. A multi-subunit chlamydia vaccine inducing neutralizing antibodies and strong IFN-γ(+) CMI responses protects against a genital infection in minipigs. Immunol Cell Biol (2016) 94:185–95. doi: 10.1038/icb.2015.79 PMC4748142 26268662

[32] Yu H Karunakaran KP Jiang X Brunham RC. Evaluation of a multisubunit recombinant polymorphic membrane protein and major outer membrane protein T cell vaccine against *Chlamydia muridarum* genital infection in three strains of mice. Vaccine (2014) 32:4672–80. doi: 10.1016/j.vaccine.2014.06.002 PMC4148050 24992718

[33] Olsen AW Theisen M Christensen D Follmann F Andersen P. Protection against chlamydia promoted by a subunit vaccine (Cth1) compared with a primary intranasal infection in a mouse genital challenge model. PloS One (2010) 5:e10768. doi: 10.1371/journal.pone.0010768 20505822 PMC2874006

