# Supplementary material for: Efficacy of Pgp3 vaccination for Chlamydia urogenital tract infection depends on its native conformation
Source: Front Immunol. 2022 Nov 16;13:1018774. doi: 10.3389/fimmu.2022.1018774 (PMC9709265; doi:10.3389/fimmu.2022.1018774)
Supplement: Supplementary file 1 [file DataSheet_1.pdf]

Supplementary Material

Supplementary Figures

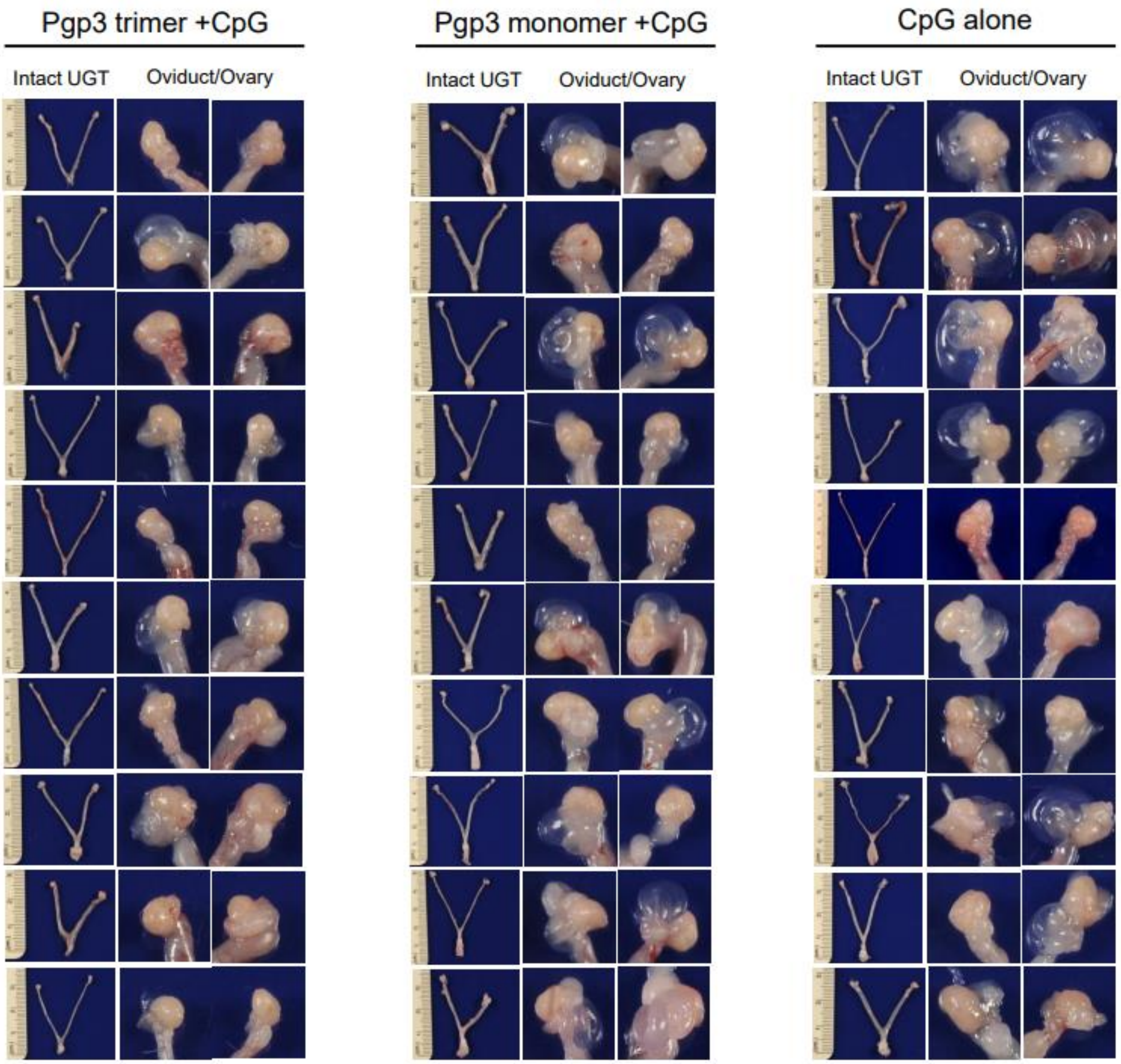

Supplementary Figure 1. Pgp3 trimer immunization reduced *C. muridarum*-induced hydrosalpinx. Three groups of mice after vaginal challenge were sacrificed for entire upper genital tract (UGT) isolation and gross pathological evaluation. The intact UGT and corresponding amplified oviduct/ovary from both left and right sides of all mice are displayed.
